# An isoform of Nedd4-2 is critically involved in the renal adaptation to high salt intake in mice

**DOI:** 10.1038/srep27137

**Published:** 2016-06-03

**Authors:** Shintaro Minegishi, Tomoaki Ishigami, Tabito Kino, Lin Chen, Rie Nakashima-Sasaki, Naomi Araki, Keisuke Yatsu, Megumi Fujita, Satoshi Umemura

**Affiliations:** 1 Yokohama City University Graduate School of Medicine, Department of Medical Science and Cardio-Renal Medicine.

## Abstract

Epithelial sodium channels (ENaCs) play critical roles in the maintenance of fluid and electrolyte homeostasis, and their genetic abnormalities cause one type of hereditary salt-sensitive hypertension, Liddle syndrome. As we reported previously, both human and rodent Nedd4L/Nedd4-2 showed molecular diversity, with and without a C2 domain in their N-terminal. Nedd4L/Nedd4-2 isoforms with a C2 domain are hypothesized to be related closely to ubiquitination of ENaCs. We generated Nedd4-2 C2 domain knockout mice. We demonstrate here that loss of Nedd4-2 C2 isoform causes salt-sensitive hypertension under conditions of a high dietary salt intake *in vivo*. The knockout mice had reduced urinary sodium excretion, osmotic pressure and increased water intake and urine volume with marked dilatation of cortical tubules while receiving a high salt diet. To the contrary, there was no difference in metabolic data between wild-type and knockout mice receiving a normal control diet. In the absence of Nedd4-2 C2 domain, a high salt intake accelerated ENaC expression. Coimmunoprecipitation studies revealed suppressed ubiquitination for ENaC with a high salt intake. Taken together, our findings demonstrate that during a high oral salt intake the Nedd4-2 C2 protein plays a pivotal role in maintaining adaptive salt handling in the kidney.

Inability to maintain normal homeostasis of body fluids and electrolytes causes various human cardiovascular diseases, including hypertension and congestive heart failure. Impaired sodium handling in tubules results in hereditary salt-sensitive hypertension. Epithelial sodium channels (ENaCs) play a pivotal role in sodium reabsorption in aldosterone-sensitive distal nephron (ASDN). The neural precursor cell expressed, developmentally down-regulated 4-like (Nedd4L/Nedd4-2) gene, rather than Nedd4-1, participates in the regulation of plasma volume and blood pressure by controlling expression of ENaCs^1^,^2^,^3^,^4^. Among various modulating proteins, **H**omologous to the **E**6-AP **C**arboxyl **T**erminus (HECT) domain-containing ubiquitin ligase, Nedd4-L/Nedd4-2, binds the PY motif of ENaC COOH terminals and catalyzes ubiquitination of the NH2 terminus of the protein for subsequent degradation. Genetic loss of ubiquitination for ENaCs by Nedd4L results in hereditary salt-sensitive hypertension, Liddle syndrome^5^. These observations shed light on the role of enhanced sodium reabsorption via ASDN through ENaCs in the development of salt sensitivity and related pathophysiological conditions, such as hypertension. Two different types of gene-targeted mouse models for Nedd4-2 gene have been generated by developmental engineering techniques and studied so far. Earlier experiments by Shi *et al.*^6^ showed that mice lacking the middle of the coding exon for Nedd4-2 had salt-sensitive hypertension and left ventricular hypertrophy as cardiovascular involvement. In contrast, another strategy used by Boase *et al.*^7^ to target terminal exons of mouse Nedd4-2 resulted in lethal respiratory distress during the perinatal period. Our initial observations indicated that both human and rat Nedd4L/Nedd4-2 had molecular diversity for alternative exon usage, generating Nedd4-2 proteins with and without C2 domain in their N-terminal^8^,^9^,^10^. As for human Nedd4L, both evolutionarily conserved and evolutionarily new C2 domains of human Nedd4L, a cryptic splice variant (rs4149601) causing a disrupted isoform product formed by a frame-shift mutation, were reported previously by Dunn and Ishigami *et al.*^9^. Genotype-phenotype correlation studies, including ours^11^, demonstrated that subjects with G allele of the variant^12^,^13^,^14^,^15^ had low-renin salt-sensitive hypertension with a high incidence of cardiovascular consequences. Using molecular biological techniques and heterologous expression systems in Xenopus oocytes, we showed dominant negative effects of evolutionarily new isoform I over constitutively expressed isoforms II and III for ubiquitination and subsequent degradation of ENaC *in vivo*^8^,^16^. Thus, the discrepant findings among past genetically engineered mice models and inherent molecular diversity with possible functional diversity of both human and rodent Nedd4-2/Nedd4L gene prompted us to further elucidate the role of Nedd4-2 gene molecular diversity in the development of salt sensitivity *in vivo*. We therefore generated mice without C2 domain of Nedd4-2 gene and performed metabolic balance studies.

## Results

### Nedd4-2 C2-knockout mice and their phenotype in general

**Figure 1:**
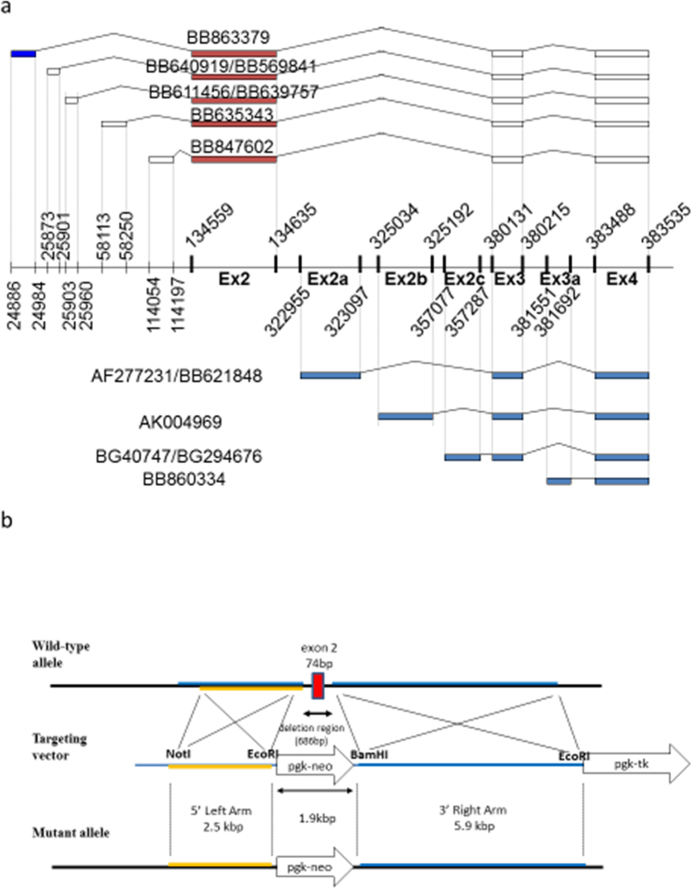
(**a**) Expressed sequence tag and cDNA alignment and results of *in silico* bioinformatic database analysis of Nedd4-2 on mouse chromosome 18q. EST sequences and cDNA sequences were defined by searching GenBank database (nr). Our analyses suggested different transcript isoforms containing C2 domain. The conserved Nedd4-2 C2 domain is encoded by exon 2. (**b**) Map of exon 2 and the targeting constructs. Genomic DNA was digested with EcoRI and BamHI and hybridization with 5′ and 3′ probes to verify the homologous recombination. Wild-type (6.4 kb) and targeted (4.1 kb) EcoRI alleles were detected by a 5′ external probe. Wild-type (12.4 kb) and targeted (8.0 kb) BamHI alleles were detected by a 3′ internal probe.

We discovered that Nedd4-2 C2 domain was encoded by exon 2 on chromosome 18 of the mouse (Fig. 1a). The targeting construct was linearized with NotI and introduced into embryonic stem (ES) cells by electroporation (Fig. 1b). Offspring from the crossing of Nedd4-2 C2 heterozygotes yielded 12.7% homozygotes, 57.7% heterozygotes, and 29.6% wild-type littermates. Neonatal death in homozygous mice did not appear to be caused by gross anatomical abnormalities. There was no significant difference between KO mice and WT littermates (Table 1).

### Radiotelemetric blood pressure measurement

**Figure 2:**
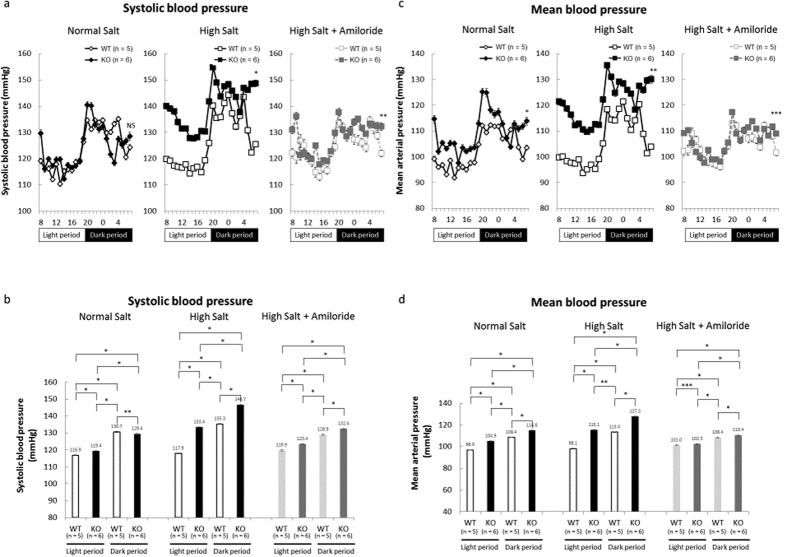
(**a**) Sodium-intake effects of wild-type and Nedd4-2 C2-knockout mice. Mean systolic blood pressure measured by radiotelemetry in wild-type (n = 5) and Nedd4-2 C2-knockout mice (n = 6). The dark bars indicate lights off (8 pm to 8 am). On a normal salt diet, systolic blood pressure did not differ between wild-type and Nedd4-2 C2-knockout mice (p = 0.0527). High salt diet caused a hypertensive response of Nedd4-2 C2-knockout mice. *p < 0.0001 compared with WTxHS. **p < 0.0001 compared with WTxHS+Amiloride. Statistical analyses were performed by two-way ANOVA for NS, HS, and HS+Amiloride. **(b)** Effects of amiloride treatment on systolic blood pressure in Nedd4-2 C2-knockout mice. Amiloride treatment significantly decreased systolic blood pressure in Nedd4-2 C2-knockout mice. *p < 0.0001. **p = 0.0058. (**c**) Comparison of 24-h average systolic blood pressure between wild-type and Nedd4-2 C2-knockout mice. *p < 0.0001 compared with WTxNS. **p < 0.0001 compared with WTxHS. ***p < 0.0001 compared with WTxHS+Amiloride. Statistical analyses were performed by two-way ANOVA on for NS, HS, and HS+Amiloride. (**d**) Comparison of mean blood pressure and amiloride treatment effects between wild-type and Nedd4-2 C2-knockout mice. *p < 0.0001. **p = 0.0001. ***p = 0.0007.

We measured systolic blood pressure (SBP), diastolic blood pressure (DBP) directly and calculated mean blood pressure (MBP), using a radiotelemetry device. Nedd4-2 C2 KO mice showed significant blood pressure elevation during a high oral salt intake, and amiloride significantly ameliorated this elevation as shown in Fig. 2a–d.

### Effects of dietary salt on water intake, urine volume, and urine sodium excretion

**Figure 3:**
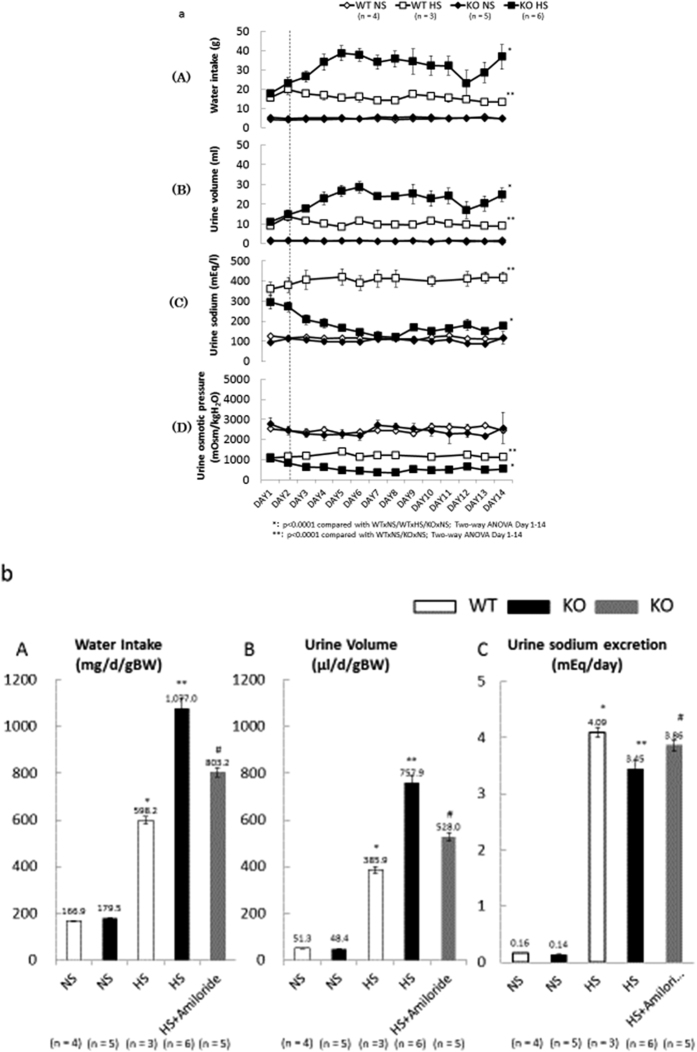
(**a**) Effects of Nedd4-2 C2-knockout on water intake (A), urine volume (B), urine sodium concentration (C), and urine osmotic pressure (D) for 14 days. Data are from normal salt wild-type mice (WT NS, n = 4), high salt wild-type mice (WT HS, n = 3), normal salt Nedd4L C2-knockout mice (KO NS, n = 5), and high salt Nedd4L C2-knockout mice (KO HS, n = 6). *p < 0.0001 compared with WTxHS, WTxNS, KOxNS. **p < 0.0001 compared with WTxNS, KOxNS. Statistical analyses were performed by two-way ANOVA on days 1–14. (**b**) Effects of amiloride treatment on water intake (A) urine volume (B) and urine sodium excretion (C) in Nedd4-2 C2-knockout mice. Data are from normal salt wild-type mice (WT NS, n = 4), high salt wild-type mice (WT HS, n = 3), normal salt Nedd4-2 C2-knockout mice (KO NS, n = 5), high salt Nedd4-2 C2-knockout mice (KO HS, n = 6), and high salt Nedd4-2 C2-knockout mice treated with amiloride (KO HS+Amiloride, n = 5). (A) *p < 0.0001 compared with WTxNS, KOxNS, KOxHS, KOxHS+Amiloride. **p < 0.0001 compared with WTxNS, KOxNS, KOxHS+Amiloride. ^#^p < 0.0001 compared with WTxNS, KOxNS. (B) *p < 0.0005 compared with WTxNS, KOxNS, KOxHS, KOxHS+Amiloride. **p < 0.0001 compared with WTxNS, KOxNS, KOxHS+Amiloride. ^#^p < 0.0001 compared with WTxNS, KOxNS. (C) *p < 0.0001 compared with WTxNS, KOxNS, KOxHS. **p < 0.001 compared with WTxNS, KOxNS, KOxHS+Amiloride. ^#^p < 0.005 compared with WTxNS, KOxNS.

There was no difference in metabolic data between KO mice and WT littermates given a normal control diet. In contrast, daily water intake and urine volume increased after 3 days of a high-salt diet in similarly treated KO mice. Urine sodium excretion remained unchanged during a normal-salt diet in KO mice and WT littermates, but it increased in KO mice after 2 days of treatment (Fig. 3a). On the basis of BW, average daily water intake on a normal-salt diet was 166.93 ± 3.17 mg/d/g BW in WT littermates and 179.52 ± 3.44 mg/d/g BW in KO mice. Average daily urine volume was 51.26 ± 1.84 μg/d/g BW in WT littermates and 48.37 ± 1.92 μg/d/g BW in KO mice. Average daily water intake and urine volume increased significantly during a high salt diet, but both of these variables were higher in KO mice (1.08 ± 0.04 g/d/g BW and 0.76 ± 0.03 g/d/g BW, respectively). Daily sodium excretion significantly decreased in KO mice given a high-salt diet. In contrast, sodium excretion was similar, with no significant differences, in KO mice and WT littermates given a normal salt intake. Treatment with amiloride significantly ameliorated reduced daily sodium excretion, but could not restore water intake and urine volume to the levels of WT littermates given a high salt diet (Fig. 3b, Table 2).

### Plasma renin and aldosterone concentrations

**Figure 4:**
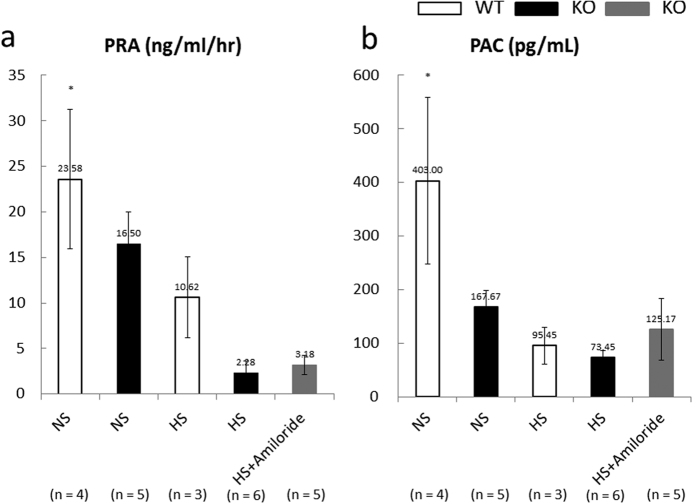
Plasma renin (**a**) and aldosterone (**b**) concentrations. Data are from normal salt wild-type mice (WT NS, n = 4), normal salt Nedd4-2 C2-knockout mice (KO NS, n = 5), high salt wild-type mice (WT HS, n = 3), high salt Nedd4-2 C2-knockout mice (KO HS, n = 6), and high salt Nedd4-2 C2-knockout mice treated with amiloride (KO HS+Amiloride, n = 5). (A) *p < 0.001 compared with KOxHS, KOxHS+Amiloride. (B) *p < 0.001 compared with WTxHS, KOxHS.

Plasma renin activity (PRA, ng/ml/hr) and plasma aldosterone concentration (PAC, pg/ml) were measured on day 14 (Fig. 4). PRA was as follows: WT NS group, 23.6 ± 7.6; KO NS group, 16.5 ± 3.5; WT HS group, 10.6 ± 4.4; KO HS group, 2.3 ± 1.3; KO HS + amiloride group, 3.2 ± 1.0. PAC was as follows: WT NS group, 403.0 ± 155.4; KO NS group, 167.7 ± 31.2; WT HS group, 95.5 ± 34.1; KO HS group, 73.5 ± 13.0; KO HS + amiloride group, 125.2 ± 57.4. Nedd4-2 C2 KO mice showed significant blood pressure elevation while receiving a high oral salt intake; however, PRA and PAC levels were suppressed. The reductions in these hormones therefore compensated to maintain normal homeostasis. No change was associated with amiloride treatment (Fig. 4).

### RNA extraction of laser-captured tubular samples and subsequent qRT-PCR analyses

**Figure 5:**
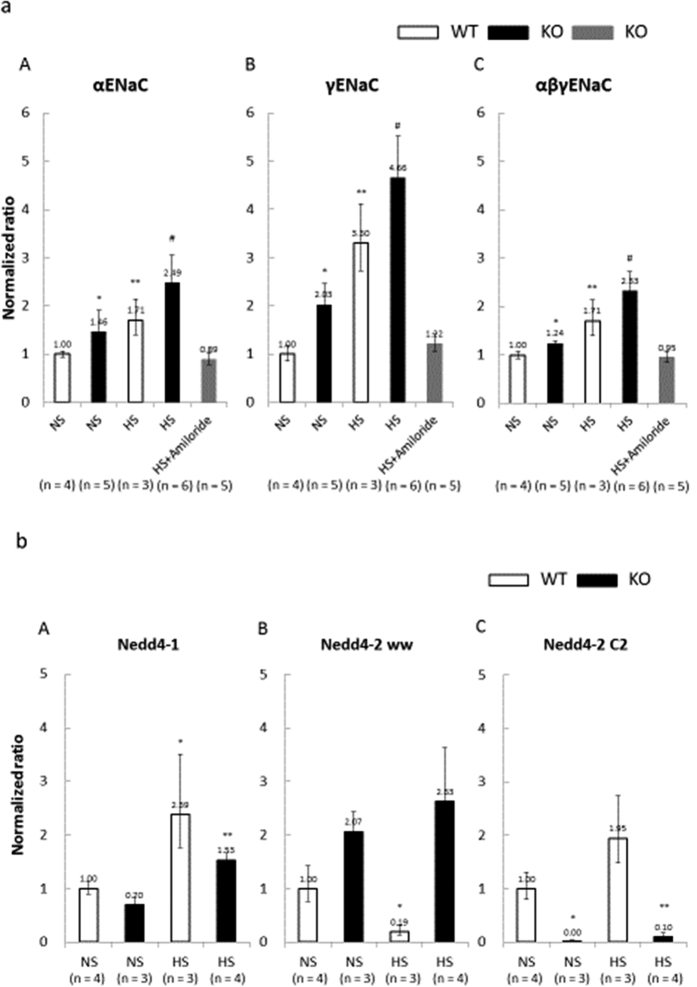
(**a**) Total RNA abundances of αENac (A) γENaC (B) and αβγENaC (C) for laser-captured DCT/CNT/CCD. Data are from normal salt wild-type mice (WT NS, n = 4), normal salt Nedd4-2 C2-knockout mice (KO NS, n = 5), high salt wild-type mice (WT HS, n = 3), high salt Nedd4-2 C2-knockout mice (KO HS, n = 6), and high salt Nedd4-2 C2-knockout mice treated with amiloride (KO HS+Amiloride, n = 5). (A) *p < 0.005 compared with WTxNS, KOxHS, KOxHS+Amiloride. **p < 0.0005 compared with WTxNS, KOxHS, KOxHS+Amiloride. ^#^p < 0.0001 compared with WTxNS, KOxHS+Amiloride. (B) *p < 0.0005 compared with WTxNS, WTxHS, KOxHS, KOxHS+Amiloride. **p < 0.0001 compared with WTxNS, KOxHS+Amiloride. ^#^p < 0.0001 compared with WTxNS, KOxHS+Amiloride. (C) *p < 0.005 compared with WTxHS, KOxHS. **p < 0.005 compared with WTxNS, KOxHS, KOxHS+Amiloride. ^#^p < 0.0001 compared with WTxNS, KOxHS+Amiloride. (**b**) Total RNA abundances of Nedd4-1 (A) Nedd4-2 ww (B) and Nedd4-2 C2 (C) for laser captured DCT/CNT/CCD. Data are from normal salt wild-type mice (WT NS, n = 4), normal salt Nedd4-2 C2-knockout mice (KO NS, n = 3), high salt wild-type mice (WT HS, n = 3), and high salt Nedd4-2 C2-knockout mice (KO HS, n = 4). (A) *p < 0.0001 compared with WTxNS/KOxNS. **p < 0.0001 compared with KOxNS. (B) *p < 0.0001 compared with WTxNS/KOxNS/KOxHS. (C) *p < 0.0001 compared with WTxNS/WTxHS/KOxHS. **p < 0.005 compared with WTxNS/WTxHS.

Kidney mRNA expression levels of α, γ and αβγENaC were measured by quantitative RT-PCR analysis of microdissected tissue samples. As compared with the WT NS group, αENaC and γENaC mRNA levels were significantly higher in the KO NS, WT HS, and KO HS groups. The total RNA expression of αβγENaC was higher in the KO HS group than in the WT NS, KO NS, and WT HS groups. The loss of Nedd4-2 C2 domain increased ENaC expression during a high-salt diet on qRT-PCR of total RNA from tissues excised by laser microdissection methods. Amiloride treatment fully abolished the increased mRNA expression of αENaC, γENaC, and αβγENaC to normal levels (Fig. 5a). We found that however Nedd4-1 and Nedd4-2 were expressed significantly in these segments of nephron as shown in Fig. 5b and those isoform products could not compensate salt handling in currently developed Nedd4-2 C2 knock out mice. Our strategies of specific gene ablation used in current experiments were validated by significantly suppressed expressions of Nedd4-2 C2 isoforms as shown in Fig. 5b.

### Semi quantitative histopathological examinations for tubular ENaCs and coimmunoprecipitation assays of βENaC and NCCT

**Figure 6:**
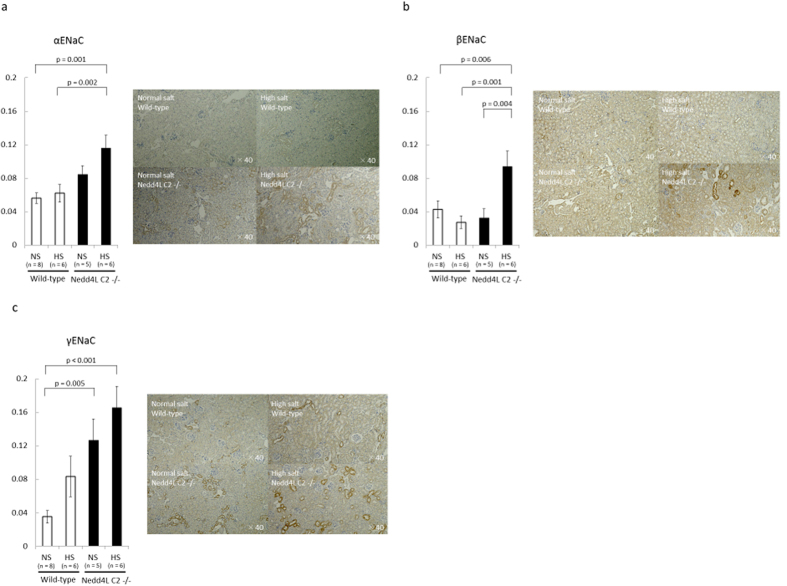
(**a**) αENaC, (**b**) ENaC, (**c**) γENaC expression by immunohistochemistry. Data are from normal salt wild-type mice (WT NS, n = 8), high salt wild-type mice (WT HS, n = 6), normal salt Nedd4-2 C2-knockout mice (KO NS, n = 5), and high salt Nedd4-2 C2-knockout mice (KO HS, n = 6). (**a**) p = 0.001 WTxNS vs KOxHS, p = 0.002 WTxHS vs KOxHS. (**b**) p = 0.006 WTxNS vs KOxHS, p = 0.001 WTxHS vs KOxHS, p = 0.004 KOxNS vs KOxHS. (**c**) p < 0.001 WTxNS vs KOxHS, p = 0.005 WTxNS vs KOxNS.

**Figure 7:**
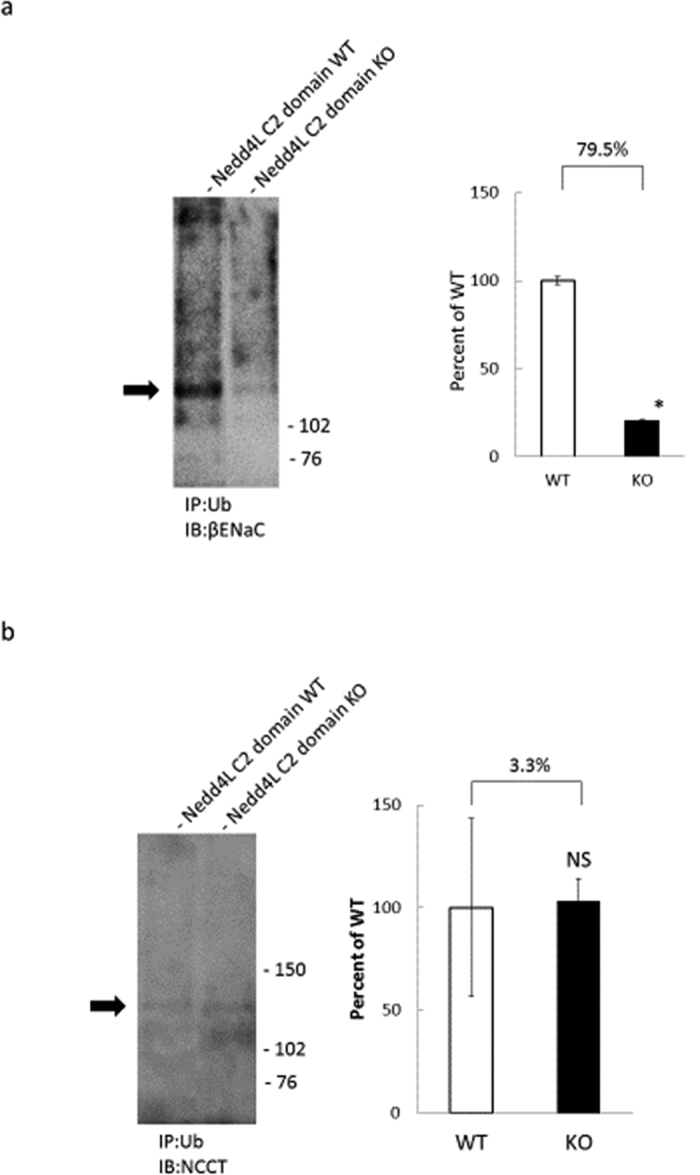
Representative findings of coimmunoprecipitation assays of βENaC (**a**), NCCT (**b**). Data are from high salt wild-type mice and high salt Nedd4-2 C2-knockout mice. *p < 0.0001 relative to WT.

Semi quantitative histopathological examinations for ENaCs in tubules revealed enhanced stained areas of each channels were significantly different between four groups as in Fig. 6a∼6c. Using immunostaining, ENaCs was observed in cortical labyrinths, in clusters of tubular sections surrounding veins, a pattern characteristic of the arcades when examined in tissue sections. It was not observed in any other tubular segment of the kidney, consistent with previous reports including ours^17^. Subsequent immunoprecipitation experiments of kidney extracts showed significantly suppressed ubiquitination for βENaC during a high-salt diet but not for NCCT(Fig. 7a, b). Taken together, these findings suggested that ubiquitinations and subsequent degradations of ENaC are suppressed in tubular epithelium of Nedd4-2 C2 KO mice under high oral sodium intake.

### Histopathological Examinations

**Figure 8:**
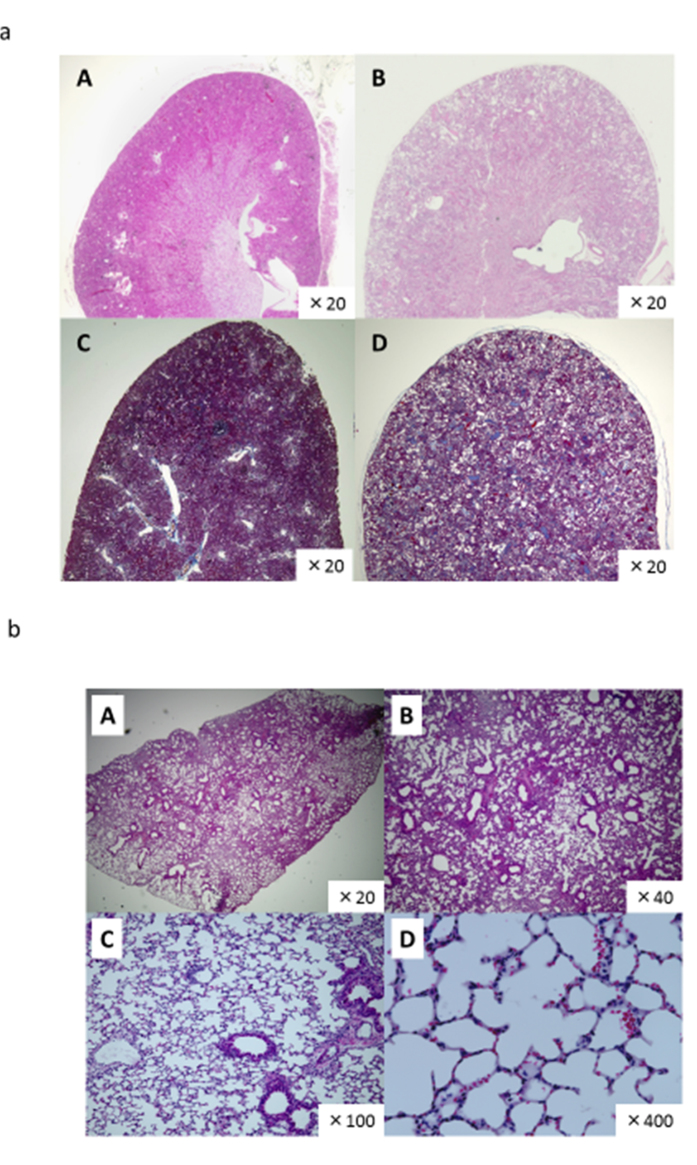
(**a**) Morphological changes in the kidney of 18 week-old wild-type (A, C) and Nedd4-2 C2-knockout (B, D) mice (by X20 magnification). Representative microscopic findings of hematoxylin eosin stain (A, B) and Masson’s trichrome stain (C, D) are shown by x20 magnification under light microscopic examinations using a BZ-9000 fluorescence microscope (Keyence, Osaka, Japan). Widespread dilation of cortical tubules was observed at week 2 in Nedd4-2 C2-knockout mice given a high salt diet. Masson-trichrome staining for serial sections showed little fibrotic changes observed in kidney of KO mice with tubular dilatation. (**b**) Representative Nedd4-2 C2-knockout (22 wks male) mouse lung. Representative Nedd4-2 C2-knockout (22wks male) mouse lung, (A) by X20 magnification, (B) by X40 magnification, (C) by X100 magnification, (D) by X400 magnification with hematoxylin eosin staining. Normal lung alveolar patterns were preserved without any accumulation of pathological surfactant in alveolus. Nedd4-2 C2 knockout mice did not show any respiratory distress during lifetime.

Representative histological examinations of kidneys showed marked dilatation of cortical tubules after 2 weeks of a high-salt diet (Fig. 8a). Masson-trichrome staining for serial sections showed little fibrotic changes observed in kidney of KO mice with tubular dilatation, suggesting that cystic changes in kidney of KO mice were not due to inflammatory process under high salt diet and hypertension. Our study showed normal lung structures with preserved alveolar pattern and without any respiratory distress during lifetime (Fig. 8b), whereas recent studies using lung epithelial Nedd4-2 KO mice revealed lung disease associated with cystic fibrosis-like changes^18^ and earlier mice Nedd4-2 KO mice to target terminal exons of mouse Nedd4-2 resulted in lethal respiratory distress during the perinatal period^7^.

## Discussion

Our studies using metabolic cages in Nedd4-2 C2 KO mice showed that higher oral salt intake promoted significant electrolyte and water imbalance with reduced daily sodium excretion, daily osmotic pressure in urine and increased daily water intake and daily urine volume, resulting in salt-sensitive hypertension. Tubular dilatation was eventually observed with marked cystic changes in the kidney. KO mice and wild littermates fed normal chow did not show any differences in phenotype such as those described above, suggesting that loss of merely one mouse Nedd4-2 isoform^9^,^10^ significantly impaired homeostatic adaptation to oral salt intake, associated with only the high-salt regimens. These findings showed that mouse Nedd4-2 with C2 domain isoform plays a critical role in regulating sodium reabsorption and homeostasis in kidney.

High oral salt intake in rural northern Japan and higher prevalences of hypertensive disease were reported in earlier periods of hypertension research^19^,^20^, and subsequent global epidemiological evidence has supported this relationship^21^. However, although higher oral salt intake significantly correlates with hypertensive disorders, individually tested dose (amount of oral salt intake)- response relationships have not been consistent^22^,^23^. For example, subjects with normal renal function were normotensive with a narrow blood pressure range despite various oral salt intakes^24^. Therefore, individuals who showed hypertension in response to a higher oral salt intake were defined as being “salt sensitive”. Higher salt intake and salt-sensitivity mutually cause hypertension and lethal cardiovascular disorders. Various attempts to reduce oral salt intake have successfully lowered nationwide blood pressure levels^25^,^26^; however, the characteristics of salt sensitivity remain to be fully elucidated, with the ultimate goal of reducing related cardiovascular morbidity and mortality^27^.

Genetic studies of human familial and hereditary hypertension by Prof. Lifton and Prof. Lalouel *et al.* in the late 20^th^ century revealed that genes encoding sodium transporters and their accessory proteins were responsible for the development of hereditary salt-sensitive disorders in human. Among various transporters, both ENaC and sodium-chloride co-transporter (NCCT) expressed along cortical tubules play critical roles in regulating and fine tuning sodium homeostasis; therefore, genetic abnormalities of ENaC and NCCT result in hereditary diseases such as Liddle syndrome and Gitelman’s syndrome.

ENaCs are ion transporters in the ASDN that play an important role in sodium reabsorption in the terminal nephron. ENaC activity in the terminal nephron is regulated by open probability, expression levels (regulation of expression), and membrane abundance (post-translational modification). ENaC is considered a major effector of aldosterone-mediated sodium reabsorption and fluid regulation. ENaC is thus a representative aldosterone inducible protein (AIP)^28^. Aberrant enhancement of ENaCs with concomitant enzymatic activation was induced by channel-activating proteases in a rodent model of salt-sensitive hypertension^29^. Post-transcriptional modifications of ENaCs by the ubiquitinating enzyme Nedd4L are also known to have an important part in the pathogenesis of Liddle’s syndrome^5^,^30^,^31^,^32^. Our studies and those of others have suggested that a common variant of the human Nedd4L gene (rs4149601) is one of the potent candidate gene variants participating in the development of human salt-sensitive hypertension and cardiovascular consequences^13^,^14^,^33^. Variants and isoform formation of the human Nedd4L gene are complex^8^,^9^, and our studies suggested that dominant negative protein-protein interactions among human Nedd4L products were key to enhancing cell surface ENaC expression *in vivo*^34^.

Developmental engineering technologies have been used to generate and examine two different types of gene-targeted mouse models for the Nedd4-2 gene, so far. Earlier experiments by Shi *et al.*^6^ showed that mice lacking the middle of the coding exon for Nedd4-2 had salt-sensitive hypertension and left ventricular hypertrophy as cardiovascular involvement. Surprisingly, another strategy targeting terminal exons of mouse Nedd4-2 by Boase *et al.*^7^ resulted in lethal respiratory distress in the perinatal period. Thus, the discrepancies between past genetically engineered mice models and the inherent molecular diversity with possible functional diversity of both human^9^ and rodent Nedd4-2 genes^10^ prompted us to further elucidate the role of the Nedd4-2 gene in the development of salt sensitivity *in vivo*.

We successfully discovered *bona-fide* exon 2 encoding C2 domain in mouse Nedd4-2 gene located at chromosome 18, using *in-silico* approaches (Fig. 1a). Our subsequent studies using Nedd4-2 C2 KO mice revealed that molecular diversity of the gene encoding Nedd4-2 was also associated with functional diversity, because deletion of merely one of the isoforms encoding Nedd4-2 with C2 domain resulted in uncompensated renal impairment with salt retention and hypertension, as described above. Our histopathological examinations revealed normal lung structures (Fig. 8b) without any respiratory distress during lifetime and we could not find significant fibrotic change and cardiac hypertrophy under light-microscopic examinations with both high and normal salt intake for Nedd4-2 KO mice currently. (data not shown) These findings together with cortical tubular specific expression in rodents^10^ suggest that Nedd4-2 with C2 domain protein plays a pivotal role in salt handling in the kidney.

Our quantitative analyses assessing gene expressions of ENaCs by laser-captured microdissected ASDN^17^ showed that higher oral salt intake with Nedd4-2 C2 protein deletions significantly enhanced gene expressions of the channels in a step-by-step manner (Fig. 5a). In addition, administration of oral amiloride fully abolished such enhanced gene expression towards normal levels, suggesting the involvement of tubular cell surface ENaCs acting as a sodium sensor and subsequent ENaC gene regulation in tubular epithelial cells. We examined expressions of ENaCs and other sodium transporters such as thiazide-sensitive sodium chloride co-transporter (NCCT), Na-K-Cl cotransporter 2 (NKCC2) in both brain and lung, and we could not find higher oral salt intake with Nedd4-2 C2 protein deletions significantly enhance gene expressions of these channels in both organ. (data not shown) This suggested that effects of oral sodium loading for the expressions of gene encoding ion-transporters were limited in kidney cortical tubular epithelium.

Finally, we successfully generated and newly identified Nedd4-2 C2 encoding exon KO mice and showed that the isoform significantly contributed to oral salt adaptations in kidney in studies. These findings suggest that Nedd4-2 with C2 protein, but not other isoforms, work as essential adaptation molecules for higher oral salt intake. Maladaptations to higher oral salt intake resulted in salt-sensitive hypertension, and this finding indicates that ENaC-Nedd4-2 C2 systems are pivotal therapeutic and pathophysiological targets for human salt-sensitive disorders such as hypertension and subsequent cardiovascular involvement. However both Shi *et al.* and Boase *et al.* adopted systemic gene ablation, our systemic Nedd4-2 C2 KO strategies and pharmacological interventions using amiloride for ENaC suppression might be major limitations in these experiments. This might be addressed by means of tissue specific gene ablation techniques for Nedd4-2 C2 isoforms and strategies other than pharmacological interventions such as *in vivo* si-RNA transfer to the tubules. Additionally, recently role of ENaC in brain for developing hypertension was proposed by Leenen *et al.*^35^,^36^, this should be evaluated in current our mouse model in future.

## Methods

### Generation of Nedd4-2 C2 domain knockout mice

Gene identification analysis of mice Nedd4-2 C2 domain coding exon was performed using BLAST and cross match program. Then, sequences of EST and cDNA in the GenBank database (nr) were aligned and compared with the sequence of mice Nedd4-2 exon 4 which was already known as transcription start site of mice Nedd4-2 without C2 domain. Cross match analyses were performed repeatedly between cDNA/EST sequences and genomic sequences, and the results were parsed with chromosome 18 of C57Bl6/J to form a consistent assembly of EST, cDNA, and genomic sequence. We synthesized targeting vector designed to disrupt exon 2 newly discovered *in silico* (Fig. 1a). The targeting construct was linearized with NotI and introduced into embryonic stem (ES) cells by electroporation (Fig. 1b). Correctly targeted ES cells were confirmed by Southern blot analysis using 5′ external probe and 3′ internal probes. Targeted ES cells were injected into blastocysts to generate chimeras. Chimeric males were mated with C57Bl/6J females, and heterozygous offspring were intercrossed to obtain mice homozygous for Nedd4-2 C2 −/−. All animal experiments were performed in accordance with the guidelines of the Animal Experiment Committee, Yokohama City University School of Medicine, and with approval of the Animal Experiment Committee, Yokohama City University School of Medicine. Nedd4-2 C2 domain genotyping was performed by polymerase chain reaction (PCR) with tail genomic DNA. Tail DNA was prepared by standard methods and DNA sequences for PCR genotyping.

### Metabolic balance studies

Age-matched (18–20 week-old) male Nedd4-2 C2 −/− mice (KO, n = 16) and wild-type littermates (WT, n = 7) were kept for 14 days in individual metabolic cages (SN-781, Shinano Manufacturing Co., Ltd., Tokyo, Japan) under controlled conditions of light, temperature, and humidity after a 5-day habituation period. They were divided into five groups and received the following diets: (1) WT given a normal-salt diet containing 0.5% NaCl (WT NS group, n = 4), (2) WT given a high-salt diet containing 8% NaCl (WT HS group, n = 3), (3) KO given a normal-salt diet containing 0.5% NaCl (KO NS group, n = 5), (4) KO given a high-salt diet containing 8% NaCl (KO HS group, n = 6), and (5) KO given a high-salt diet containing 8% NaCl plus amiloride (KO HS + amiloride group, n = 5). Amiloride (Biomol International, LP, Plymouth Meeting, PA, USA) was administered daily by oral gavage at a dose of 1 mg/kg per day. In a previous study, suitable drug concentrations of drinking water were determined^17^. Body weights (BW) and water intake were measured, and urine was collected daily. Urine sodium concentrations were measured using specific electrodes (Oriental Yeast Co., Ltd., Tokyo, Japan). Urine osmolality was determined using a cryoscopic osmometer (Oriental Yeast Co., Ltd., Tokyo, Japan). Urine creatinine concentrations were measured by enzyme-linked immunosorbent assay (ELISA). On day 14, the mice were anesthetized with diethyl ether, and blood samples were collected by cardiac puncture. Plasma renin and aldosterone concentrations were measured by radioimmunoassay kits, using Renin-Riabead (Dinabot, Tokyo, Japan) and SPAC-S Aldosterone (TFB, Tokyo, Japan), respectively. Organs were dissected and fixed for subsequent total RNA extraction, protein extraction, immunoprecipitation, and histological analyses.

### Blood pressure measurements by radiotelemetry device

For further evaluation of cardiovascular function, direct blood pressure was measured by radiotelemetry (HD-X11, Primetech, Tokyo, Japan). Male Nedd4-2 C2 −/− mice (n = 6; 14–16 weeks old) and wild-type littermates (n = 5; 14–16 weeks old) were used for this experiment. Mice were anesthetized, and a pressure-sensing catheter was implanted into the left carotid artery. After the surgical recovery period, each mouse was housed individually under a 12-hour light-dark cycle. Systolic blood pressure (SBP) and diastolic blood pressure (DBP) were recorded every minute by the device. Basal blood pressure was measured for 5.6 ± 0.8 days for KO mice, and 5.4 ± 1.0 days for WT mice, respectively, on a normal-salt diet, after which the mice received a high-salt diet for 5.6 ± 0.8 days for KO mice, and 5.4 ± 1.0 days for WT mice, respectively. For amiloride treatment, all mice received amiloride by daily intraperitoneal injection (1 mg/kg) for 4.3 ± 0.4 days for KO mice, and 4.2 ± 0.5 days for WT mice, respectively.

### MGB probes for quantitative RT-PCR analyses

We used TaqMan probes for mouse αENaC (Mm00803386_m1), and γENaC (Mm00441228_m1), mNedd4-1(Mm00441228_m1), mNedd4-2(Mm00441228_m1), all of which were purchased from Applied Biosystems. The αβγENaC probes have been designed to mix TaqMan probes for α, β, and γ subunits (Mm00803386_m1, Mm00441215_m1, and Mm00441228__m1), respectively. Primers specific for mouse Nedd4-2 C2 domain were synthesized according to the sequences above mentioned.

### RNA isolation and quantitative RT-PCR analyses

Total RNA abundances of α, γ, αβγ ENaCs, Nedd4-1, Nedd4-2 WW, and Nedd4-2C2 in laser-microdissected late distal tubules/connecting tubules/cortical collecting ducts (late DCT/CNT/CCD) tubules were measured by quantitative reverse transcriptase (qRT)-PCR analysis. Kidney sections stained with anti-Calbindin D28K antibodies were used as a marker of late DCT/CNT/CCD. Specific regions, expressing Calbindin D-28K, were carefully microdissected using Leica CTR 6000 (Leica Microsystems, Wetzlar, Germany) and collected into the cap of a 0.2-ml microtube. After microdissection, tissues were pooled together for total RNA extraction using an RNeasy FFPE Kit (Qiagen, Hilden, Germany). Synthesis of cDNA from total RNA was carried out using High Capacity RNA-to-cDNA Master Mix (Applied Biosystems, Foster City, Calif., USA). All PCRs were performed three times in duplicate.

### Semi quantitative histopathological examinations for tubular ENaCs

The stained area of ENaCs with anti-α, β, and γENaC antibodies (kindly provided by Dr. Kitamura, and Dr. Kakizoe, Kumamoto University) in each DCT/CNT/CCD tubule selected was calculated using a computerized touch pen device under a BZ9000 microscope with Dynamic Cell Count BZ-HIC image analysis software (Keyence, Osaka, Japan) as previously reported^17^. Calculations were performed six times by two independent observers who were blinded to the details of the experiment.

### Coimmunoprecipitation experiments for βENaC and NCCT with anti-ubiquitin monoclonal antibody

To confirm the ubiquitination of ion transporters, coimmunoprecipitation assays were performed using a Dynabeads Co-immunoprecipitation Kit (14321D, Invitrogen, Carlsbad, CA, USA). Kidney extracts were prepared according to the manufacturer’s protocol. After removing debris and adjusting the protein concentrations, tissue lysates were incubated with Dynabeads coupled with anti-ubiquitin monoclonal antibody (sc-271289, Santa Cruz Biotechnology, Santa Cruz, CA, USA) at 4 °C for 30 min. Normal mouse IgG (sc-2025, Santa Cruz Biotechnology, Santa Cruz, CA, USA) served as a control for co-immunoprecipitation. The beads were washed, and bound proteins were eluted in SDS-PAGE sample buffer. Western blot with anti-βENaC polyclonal and anti-NCCT polyclonal antibodies (Chemicon International Inc, Temecula, CA, USA) was then performed.

### Histopathological examinations

For histological examinations, mouse tissues were fixed in 4% paraformaldehyde, embedded in paraffin, and sliced into sections (4 μm thick). The sections were stained with hematoxylin-eosin (HE) and Masson’s trichrome stain by standard methods. Morphometric analysis was performed using a BZ-9000 fluorescence microscope (Keyence, Osaka, Japan).

### Statistical Analyses

Data are expressed as means ± SE. Statistical analyses were performed with SPSS software (Dr. SPSS, SPSS Japan, Tokyo, Japan) and StatView (ver. 5, SAS, Cary, NC, USA). The relative abundance values of mouse transcripts and proteins in the kidneys were compared statistically by repeated measures analysis of variance (ANOVA) followed by Bonferroni multiple comparison tests. P < 0.05 was considered to indicate statistical significance.

## Additional Information

**How to cite this article**: Minegishi, S. *et al.* An isoform of Nedd4-2 is critically involved in the renal adaptation to high salt intake in mice. *Sci. Rep.* **6**, 27137; doi: 10.1038/srep27137 (2016).

## Figures and Tables

**Table 1:** Morphological data in wild-type and Nedd4-2 C2-knockout mice.

	Wild-type		Nedd4-2 C2 −/−		p (ANOVA)
	NS (n = 4)	HS (n = 3)	NS (n = 5)	HS (n = 6)	
BW on day 0 (g)	27.23 ± 0.73	26.47 ± 0.96	28.26 ± 0.58	28.27 ± 0.63	0.296
BW on day 14 (g)	27.58 ± 0.45	26.53 ± 1.43	28.10 ± 0.34	28.65 ± 0.43	0.161
%LKW/BW on day 14	0.58 ± 0.03	1.07 ± 0.14^a^	0.67 ± 0.05	0.84 ± 0.08	0.007
%HW/BW on day 14	0.48 ± 0.04	0.61 ± 0.04	0.56 ± 0.03	0.72 ± 0.06^b^	0.029

Data are expressed as means  ±  SE. BW, body weight; LKW, light kidney weight; HW, heart weight.

^a^p < 0.05 vs WTxNS, KOxNS; ^b^p < 0.05 vs WTxNS.

**Table 2:** Metabolic parameters in wild-type and Nedd4-2 C2-knockout mice.

	Wild-type		Nedd4-2 C2 −/−			p (ANOVA)
	NS (n = 4)	HS (n = 3)	NS (n = 5)	HS (n = 6)	HS+Amiloride (n = 5)	
Water intake (g)	4.58 ± 0.09^acd^	15.89 ± 0.49^bcd^	5.05 ± 0.10^cd^	30.71 ± 1.30^d^	23.74 ± 0.61	<0.0001
Urine volume (ml)	1.40 ± 0.05 ^abcd^	10.26 ± 0.41^bcd^	1.36 ± 0.06^cd^	21.53 ± 0.95^d^	15.59 ± 0.54	<0.0001
Urine Osm (mOsm/kgH2O)	2481 ± 35^acd^	1191 ± 35^bcd^	2421 ± 64^cd^	574 ± 31^d^	911 ± 23	<0.0001
Urine Na (mEq/l)	50.30 ± 0.73^acd^	174.35 ± 4.55^bcd^	44.27 ± 1.16^cd^	78.24 ± 3.72^d^	112.63 ± 2.79	<0.0001
Urine K (mEq/l)	317.98 ± 5.17 ^abcd^	64.52 ± 1.61^bcd^	344.06 ± 9.72^cd^	32.44 ± 2.32	29.49 ± 2.02	<0.0001
Urine Cr (mg/dl)	22.71 ± 0.45 ^abcd^	5.50 ± 0.26^bcd^	22.08 ± 0.74^cd^	0.15 ± 0.25	2.76 ± 0.21	<0.0001
Urine Na excretion (mEq/day)	0.16 ± 0.01^acd^	4.09 ± 0.09^bc^	0.14 ± 0.01^cd^	3.45 ± 0.16^d^	3.86 ± 0.11	<0.0001
Urine K excretion (mEq/day)	0.44 ± 0.01^ac^	0.65 ± 0.02^bd^	0.45 ± 0.02^c^	0.59 ± 0.02^d^	0.42 ± 0.02	<0.0001
Urine Na/Cr ratio	2.25 ± 0.05^acd^	32.78 ± 1.14^bd^	2.09 ± 0.07^cd^	28.50 ± 1.01^d^	50.40 ± 2.55	<0.0001

Data are expressed as means ± SE. Urine samples were collected once daily for up to 14 days.

Osm, osmotic pressure; Na, sodium; K, potassium; Cr, creatinine.

^a^p < 0.01 vs WTxHS; ^b^p < 0.01 vs KOxNS; ^c^p < 0.01 KOxHS; ^d^p < 0.01 KOxHS+Amiloride.
